# Ethanolic Extract of *Artemisia vulgaris* Leaf Promotes Apoptotic Cell Death in Non-Small-Cell Lung Carcinoma A549 Cells through Inhibition of the Wnt Signaling Pathway

**DOI:** 10.3390/metabo13040480

**Published:** 2023-03-27

**Authors:** Rohit Kumar Tiwari, Afza Ahmad, Ahamad Faiz Khan, Lamya Ahmed Al-Keridis, Mohd Saeed, Nawaf Alshammari, Nadiyah M. Alabdallah, Irfan Ahmad Ansari, Farina Mujeeb

**Affiliations:** 1 Department of Biosciences, Integral University, Lucknow 226026, Uttar Pradesh, India; 2 Department of Bioengineering, Integral University, Lucknow 226026, Uttar Pradesh, India; faizkhan@iul.ac.in; 3 Biology Department, Faculty of Science, Princess Nourah Bint Abdulrahman University, P.O. Box 84428, Riyadh 11671, Saudi Arabia; 4 Department of Biology, College of Sciences, University of Hail, P.O. Box 2440, Hail 34464, Saudi Arabia; 5 Department of Biology, College of Science, Imam Abdulrahman Bin Faisal University, P.O. Box 1982, Dammam 31441, Saudi Arabia; 6 Basic & Applied Scientific Research Centre, Imam Abdulrahman Bin Faisal University, P.O. Box 1982, Dammam 31441, Saudi Arabia

**Keywords:** *Artemisia vulgaris*, non-small-cell lung carcinoma, oxidative stress, apoptosis, A549 cells, Wnt signalling pathway

## Abstract

The Wnt signaling pathway is reported to be associated with lung cancer progression, metastasis and drug resistance, and thus it is an important therapeutic target for lung cancer. Plants have been shown as reservoirs of multiple potential anticancer agents. In the present investigation, the ethanolic leaf extract of *Artemisia vulgaris* (*AvL*-EtOH) was initially analyzed by means of gas chromatography-mass spectrometry (GC–MS) to identify the important phytochemical constituents. The GC–MS analysis of *AvL*-EtOH exhibited 48 peaks of various secondary metabolites such as terpenoids, flavonoids, carbohydrates, coumarins, amino acids, steroids, proteins, phytosterols, and diterpenes. It was found that the treatment with increasing doses of *AvL*-EtOH suppressed the proliferation and migration of lung cancer cells. Furthermore, *AvL*-EtOH induced prominent nuclear alteration along with a reduction in mitochondrial membrane potential and increased ROS (reactive oxygen species) generation in lung cancer cells. Moreover, *AvL*-EtOH-treated cells exhibited increased apoptosis, demonstrated by the activation of caspase cascade. *AvL*-EtOH also induced downregulation of Wnt3 and β-catenin expression along with cell cycle protein cyclin D1. Thus, the results of our study elucidated the potential of bioactive components of *Artemisia vulgaris* in the therapeutic management of lung cancer cells.

## 1. Introduction

Lung cancer is the most common carcinoma and the main cause of cancer-associated morbidity globally. Lung cancer contributes 11.4% to the global cancer burden, with an estimated 2,206,771 new cases in 2020. Furthermore, the recent report of Global Cancer Observatory also stated that lung cancer resulted in the death of 1,796,144 individuals, which constituted 18% of the 9,958,133 deaths reported from different types of cancer [1]. Histologically, lung cancer patients are broadly differentiated as non-small cell lung carcinoma (NSCLC) and small cell lung carcinoma (SCLC), where around 80% of all lung cancer cases are of the NSCLC type [2]. Patients suffering from lung carcinomas are commonly diagnosed at advance stages, resulting in a reduced 5-year survival rate of merely 56%. Intriguingly, the 5-year survival rate of lung cancer patients is relatively lower than that of various other cancers [1]. The standard clinical management of lung cancer is restricted by the intrinsic toxicities with associated resistance of frontline chemotherapeutics [3,4]. Additionally, in patients diagnosed with advance stages of lung cancer, chemotherapeutics fail to exhibit considerable effects, which subsequently results in the failure of treatment in nearly 90% of related cases [5]. To overcome these specific drawbacks of current treatment, a surge can be observed in exploring traditional herbal medicine for treating lung carcinomas. It is believed that the broad pharmacological effect of traditional medicines exerts multiple beneficial effects on different molecular targets without any considerable side-effects [6,7]. Therefore, it is believed that the development of such plant based traditional medicines may lead to the identification of several novel active ingredients and combinations that can exert desired effects on different signaling pathways involved in cancer, with concomitant amelioration of side-effects in comparison with standard chemotherapeutics.

Wnt signaling is an established regulator of cell migration, differentiation, apoptosis and cell proliferation of nearly every tissue in the body. Homeostatic Wnt signaling is a critical regulator of developing embryo and is also a prerequisite for the balance of new and dying cells in an adult. Mutated or abrupt expression of Wnt signaling is frequently associated with debilitating diseases including cancer. In several carcinomas, it is reported that hyper-activation of Wnt/β-catenin signaling is closely related to the onset of metastasis, invasion, and migration, as well as the development of resistance against chemotherapeutics. Importantly, patients diagnosed with lung cancer exhibit varying expression levels of key molecules involved in Wnt/β-catenin signaling including cyclinD1, Wnt1, SOX-2 along with β-catenin [8].

*Artemisia vulgaris*, also referred to as mugwort, is a member of the Asteraceae family, and this family comprises several hundred species distributed worldwide. This plant is traditionally used as an ethnopharmacological agent in various health conditions, such as gastrointestinal disorders, infertility, menopausal and menstrual problems, nosebleeds, headaches, epilepsy, muscle spasms, gout, circulatory problems, rheumatism, fever, asthma, and contact dermatitis [9]. It has long been reported that the genus *Artemisia* is a source of an important bioactive compound called as artemisinin. Its derivatives have also been regarded as propitious cancer therapeutics that affect various signaling cascades. Their plausible mechanism of action involves the obstruction of the cell cycle, apoptosis induction and reduced proliferation rate [10]. The anticancer efficacy of the methanolic extract of the aerial part of this plant has been explored on a range of human cancer cell lines such as MCF-7 (breast cancer), HeLa (cervical cancer), A7R5 and 293 T (normal cell lines) [11]. Furthermore, another report demonstrated that the essential oil of the plant obtained from the leaves and buds inhibited the proliferation of human HL-60 promyelocytic leukemia cell line [12]. An earlier study performed in 2020 established the genotoxic and cytotoxic properties of the methanolic extracts of *A. vulgaris* [13]. However, the underlying mechanism of the antiproliferative and apoptotic potential of ethanolic extract of *A. vulgaris* (*AvL*-EtOH) against the A549 NSCLC cell line has largely remains un-deciphered so far. In this study, we investigate the antiproliferative and apoptotic efficacy of the *AvL*-EtOH in an in vitro model of human NSCLC cells.

## 2. Materials and Methods

### 2.1. Materials

Hoechst-33342, propidium iodide (PI), and 2,7-dichlorodihydrofluorescein diacetate (DCFH-DA) were purchased from Sigma (St. Louis, MO, USA). Cell culture–related consumables, namely Dulbecco’s modified Eagle medium (DMEM)-high-glucose, fetal bovine serum (FBS), antibiotic–antimycotic solution, 3-(4,5-dimethylthiazol-2-yl)-2,5-diphenyl tetrazolium bromide (MTT) RNase A, and HiPurATM Total RNA Miniprep Purification Kit were obtained from HiMedia, Mumbai, India. The Verso cDNA Synthesis Kit and DyNAmoColorFlash SYBR Green Quantitative Polymerase Chain Reaction (qPCR) Kit were procured from Thermo-Scientific, Waltham, MA, USA. The Mitochondrial Membrane Potential (ΔΨm) Assay Kit was obtained from G-Biosciences, St Louis, MO, USA. The primer sequences for the PCR-based analysis were obtained from IDT, Coralville, IA, USA. The ethanol and Soxhlet apparatus used in the study were procured from Merck (Branchburg, NJ, USA) and Sigma-Aldrich, St Louis, MO, USA.

### 2.2. Methods

#### 2.2.1. Plant Sample and Extract Preparation

The leaves of *Artemisia vulgaris* L. were collected from the mountains of Malagiri, a village in Gulmi District in the Lumbini Zone of Central Nepal. The plant specimen was authenticated by Dr. Muhammad Arif, Department of Pharmacognosy and Photochemistry, Faculty of Pharmacy, Integral University, Lucknow, with voucher specimen ref. no: IU/PHAR/HRB/22/07. 

Initially, *A. vulgaris* leaves were washed and dried in the shade for at least a week. Subsequently, they were ground by using a pestle and mortar. *AvL*-EtOH was prepared according to a previous study [11]. Briefly, the *A. vulgaris* leaf powder was processed through the Soxhlet extraction process by using 70% ethanol (5 h; 60–65 °C) followed by 2 h of sonication. The extract was filtered through Whatman paper no.40 and the resultant filtrate was evaporated using a rotary vacuum evaporator. Thereafter, the dried extract was dissolved in DMSO. This was again filtered and stored at 4 °C, and was subsequently used for biological assays.

#### 2.2.2. Cell Culture

Human NSCLC A549 cells were acquired from the National Centre for Cell Sciences, Maharashtra, India, cell repository. These cells were maintained in the culture facility of IIRC-1, Integral University, by using DMEM-high-glucose media complemented with FBS (10% *v*/*v*) and an antibiotic–antimycotic solution (1% *v*/*v*) in a controlled humidified atmosphere with 5% CO_2_.

#### 2.2.3. GC-MS Analysis

The gas chromatography-mass spectrometry (GC-MS) analysis of ethanolic extracts of A. vulgaris leaves was carried out by using a GC-MS (Model; QP 2010 Plus, Shimadzu, Tokyo, Japan) equipped with aVF-5ms fused silica capillary column 30 m in length, 0.25 mm in diameter, and 0.25 μm in film thickness. The column oven temperature was programmed from 80 °C to 300 °C for 2 °C min^−1^. Ionization of the sample components was performed in electron impact mode (EI, 70 eV). The temperature of the injector was set to 260 °C and the that of the detector was set to 230 °C. Pure helium gas (99.99%) was used as a carrier gas fixed with a flow rate of 1.21 mL/min. The mass range from 40 to 650 *m*/z was scanned at a rate of 3.0 scans/s. Then, 2.0 µL of the of ethanolic extract of *A. vulgaris* was injected with a Hamilton syringe into the GC-MS manually for total ion chromatographic analysis, employing the split injection technique. The total running time of GC-MS was 54 min. The relative percentage of extract constituent was expressed as a percentage with peak area normalization. The bioactive compounds of the extract were identified by comparing their retention indices and patterns of mass spectra with reference to the database of the National Institute Standard and Technology (NIST), having more than 62,000 patterns. The spectrum of the unknown component was compared with the spectrum of the known components present in the NIST library. 

#### 2.2.4. Cell Viability Assay

The cytotoxic effects of *AvL*-EtOH on the NSCLC A549 cells were investigated using the MTT assay, as previously described [14]. Initially, 5 × 10^3^ A549 cells per well of a 96-well plate were subjected to 100, 200, and 400 μg/mL *AvL*-EtOH treatment for 24 h. Then, the cells were exposed to 5 mg/mL MTT dye (10 μL/well) and incubated for another 4 h. Eventually, the formazan crystal was solubilized (100 μL dimethyl sulfoxide per well), and the cellular viability was estimated as a percentage by using the following formula:

Cell viability percentage (%) = (Mean absorbance at 570 nm − Mean absorbance at 630 nm) treatment/(Mean absorbance at 570 nm − Mean absorbance at 630 nm) control × 100

#### 2.2.5. Morphological Evaluation

The A549 cells were examined for morphological alterations after treatment with *AvL*-EtOH for 24 h as compared to untreated controls. A total of 1 × 10^4^ A549 cells/well of a 96-well plate were exposed to the concentrations mentioned above of *AvL*-EtOH for 24 h. After that, cells were examined for morphological alterations using FLoid Cell Imaging Station, Thermo-Scientific, Waltham, MA, USA.

#### 2.2.6. Colony Formation Assay

As stated previously, the colony formation assay was performed with slight modifications [15]. In total, 1 × 10^3^ A549 cells were transferred to each well of a six-well plate and treated with the aforementioned *AvL*-EtOH concentrations. The cells were then incubated at 37 °C in a CO_2_ incubator for at least 7 days for colony formation. Finally, the colonies were washed with 1× phosphate-buffered saline (PBS) and stained with 0.1% crystal violet, and then the colonies were counted manually. 

#### 2.2.7. Hoechst/PI Staining

*AvL*-EtOH was further evaluated for its apoptosis-inducing potential in the A549 cells through Hoescht-33342 and PI staining. In total, 1 × 10^3^ A549 cells seeded in a 96-well plate were subjected to different *AvL*-EtOH concentrations (100, 200, and 400 μg/mL) for 24 h and exposed to Hoescht-33342 (30 min) followed by PI (30 min). Subsequently, the cells were visualized on blue (excitation: emission = 390/40:446/33 nm) and red (excitation: emission = 586/15:646/68 nm) filters of the FLoid Cell Imaging Station.

#### 2.2.8. Measurement of Caspase-8, -9, and -3 Activity

To determine the activities of caspase-8, -9 and -3 in human lung cancer cells, a colorimetric kit was used in accordance with the manufacturer’s protocol. Post treatment with *AvL*-EtOH (concentrations as stated), 3 × 10^6^ A549 cells seeded in a 96-well plate were lysed using a chilled lysis buffer (50 μL) along with 10 min incubation on ice. The resultant cell suspension was centrifuged (10,000× *g* for 1 min; 4 °C), and the supernatant was collected and placed on ice. Thereafter, 50 μL/well of the lysate was added into a 96-well plate, along with 50 μL of reaction buffer consisting of DTT (10 mM). Subsequently, IETD-pNA, LEJD-pNA and DEVD-pNA substrates (4 mM) specific to caspase-8, -9 and -3 were added into each well and the plate was further incubated for 10 min. At the end of incubation, the absorbance of the plate was recorded using a microtiter plate reader at 405 nm. The percentage (%) increase in the caspases’ activity was determined by comparing the result with the level of untreated A549 cells as the control. 

#### 2.2.9. Mitochondrial Membrane Potential Quantification

Alterations in the ΔΨm of the *AvL*-EtOH-treated A549 cells were investigated through microscopy and flow cytometry. For the microscopic assessment of altered ΔΨm, 1 × 10^3^ A549 cells seeded in a 96-well plate were treated with *AvL*-EtOH (at the stated concentrations for 24 h) followed by JC-1 treatment (2 μM; 30 min) and subsequently visualized on red and green filters of the FLoid Cell Imaging Station. For flow cytometry, 1 × 10^5^ cells were exposed to varying *AvL*-EtOH concentrations (aforementioned) for 24 h in a 6-well plate. Then, the cells were washed twice with 1X PBS and incubated with JC-1 dye (2 μM) for 30 min in the dark; subsequently, the fluorescence of JC-1 was quantified using a flow cytometer.

#### 2.2.10. Determination of Intracellular Reactive Oxygen Species Levels

The intracellular reactive oxygen species (ROS) level was assessed using DCFH-DA dye, as described previously [16]. In total, 1 × 10^3^ A549 cells per well of a 96-well plate were exposed to varying *AvL*-EtOH concentrations for 24 h under optimum culture conditions. Then, the cells were re-exposed to 25 μM DCFH-DA in the dark for 30 min. Finally, the fluorescence within cells was visualized qualitatively for alterations within the levels of DCF-DA-mediated green fluorescence (FLoid Cell Imaging Station).

For flow cytometric evaluations, the same process was repeated with 1 × 10^5^ cells seeded in a 6-well plate with slight modifications. After DCFH-DA exposure, the cells were detached and pelleted at 4 °C through centrifugation (1500 rpm; 2 min). Subsequently, the pellets were resuspended in 1X PBS before their mean fluorescence intensity was evaluated through the FITC channel of FACS Calibur (BD Biosciences, Franklin Lakes, NJ, USA).

#### 2.2.11. Quantification of Apoptosis 

As reported previously, apoptosis was measured based on phosphatidylserine externalization using a flow cytometer with slight modifications [17]. Approximately 1 × 10^5^ A549 cells seeded in a 6-well plate were cultured with 100, 200, and 400 μg/mL of *AvL*-EtOH for 24 h. The cells were harvested, washed with chilled PBS, and suspended in 100 μL of 1X binding buffer. Furthermore, the cells were exposed to Annexin-V/FITC (5 μL) and PI (5 μL) in the dark (30 min) and, after that, analyzed using a flow cytometer (BD Biosciences, Franklin Lakes, NJ, USA).

#### 2.2.12. DNA Damage Assay

The quantity of 8-hydroxy-29-deoxyguanosine (a by-product of DNA damage) was determined in the *AvL*-EtOH-treated A549 cells for 24 h by using the OxiSelectTM Oxidative DNA Damage Enzyme-Linked Immunosorbent Assay (ELISA) Kit (Cell Biolabs, Inc., San Diego, CA, USA), according to the manufacturer’s instructions.

#### 2.2.13. Quantification of Cytosolic Cytochrome-c Levels

The cytosolic levels of cytochrome-c were quantified using a commercially available ELISA kit following the manufacturer’s instructions [18]. Approximately 1 × 10^6^ A549 cells were exposed to varying concentrations (100, 200, and 400 µg/ mL) of *AvL*-EtOH for 24 h. Subsequently, the A549 cells from different treatment groups were centrifugated for 10 min (37 °C; 1000 rpm). After that, the pellet of each group was washed with ice-cold PBS. The cytosolic protein content was extracted from the pellet through a T-PER reagent (Thermo-Fischer Scientific, Waltham, MA, USA). Eventually, the cytosolic cytochrome-c levels in the different treatment groups were quantified and compared with those of the control using the KHO1051 ELISA kit (Thermo-Fischer Scientific, Waltham, MA, USA), according to the manufacturer’s instructions. 

#### 2.2.14. Quantification of PARP Cleavage

The levels of cleaved PARP in the A549 cells exposed to varying *AvL*-EtOH concentrations (100, 200, and 400 µg/ mL) for 24 h were determined using the PARP Cleaved [214/215] ELISA kit (Thermo-Fischer Scientific, USA). The absorbance of each treatment group was read at 450 nm on a microplate reader and compared with that of the untreated A549 cells.

#### 2.2.15. Reverse Transcriptase qPCR Evaluations

In total, 1 × 10^7^ A549 cells seeded in a 6-well plate were subjected to *AvL*-EtOH (100, 200, and 400 μg/mL) treatment for 24 h, and then, the total RNA was extracted using a HiPurATM Total RNA Miniprep Purification Kit, Himedia, India (Catalogue No: MB602) following the manufacturer’s protocol. Furthermore, as per the manufacturer’s instructions, the Verso cDNA synthesis kit, Thermo Scientific, USA (Catalogue No: AB1453A) was used to prepare cDNA synthesized from 2 μg of isolated RNA. The primers used in this study are listed in Table 1. Subsequently, the PCR assay was performed using the DyNAmoColorFlash SYBR Green qPCR Kit according to the manufacturer’s instructions.

#### 2.2.16. Statistical Analysis

Data are presented as the mean ± standard error of the values obtained in three discrete experiments performed in triplicate. The statistical significance among different treatment groups as compared with the untreated control was determined through one-way analysis of variance followed by the Dunnett post hoc test. A *p*-value of <0.05 was considered statistically significant. The levels of significance were * *p* < 0.05, ** *p* < 0.01, and *** *p* < 0.001. 

## 3. Results

### 3.1. GC-MS Analysis of Ethanolic Extract of A. vulgaris

**Figure 1:**
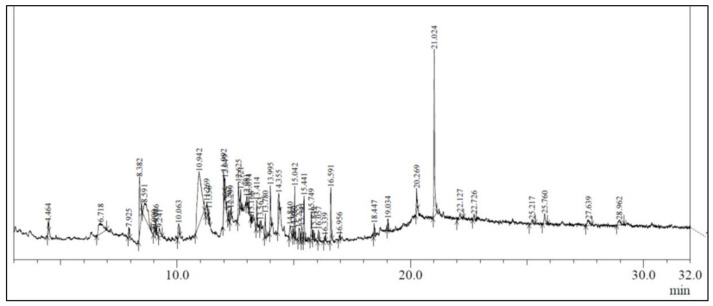
GC-MS chromatogram of the ethanolic extract of *Artemisia vulgaris*.

The ethanolic leaf extract of *A. vulgaris* L. showed the presence of several bioactive compounds (Figure 1), which are listed in Table 2.

In the present study, the investigation of ethanolic extracts of *A. vulgaris* leaves revealed the presence of multiple phytoconstituents such as terpenoids, flavonoids, carbohydrates, coumarins, amino acids, steroids, proteins, phytosterols, and diterpenes. The GC–MS chromatogram of *A. vulgaris* leaves revealed the presence of 48 phytochemical compounds which could contribute to the medicinal properties of this plant species. An earlier published report also demonstrated the presence of eudesmane-type sesquiterpene, luteolin, morin, triterpenes, flavonoids, coumarin, and eriodictyol [19,20,21,22,23,24].

### 3.2. AvL-EtOH Inhibits the Cell Viability of A549 Cells

**Figure 2:**
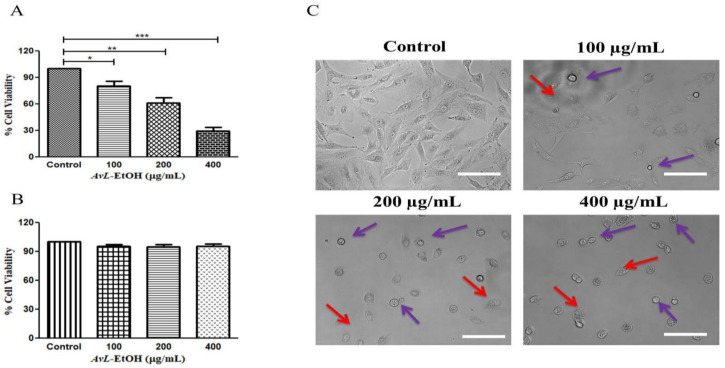
Effect of *AvL*-EtOH at different concentrations (100, 200 and 400 µg/mL) against (**A**) A549 cells. (**B**) Insignificant cytotoxic effects of *AvL*-EtOH against normal human lung fibroblast WI38 cells (**C**) Morphological alterations within A549 cells post treatment with *AvL*-EtOH. * *p* < 0.05, ** *p* < 0.01 and *** *p* < 0.001. Scale bar= 100 µm.

Initially, we aimed to elucidate the cytotoxic potential of *AvL*-EtOH on A549 cells at different concentrations of 100, 200 and 400 µg/mL. We calculated IC50 (164.6 ± 2.21 µg/mL) on the basis of these above-mentioned concentrations. The findings of the MTT assay demonstrated that *AvL*-EtOH (100, 200, and 400 μg/mL) substantially inhibited the viability of the A549 cells, which were found to be 84.42 ± 2.71%, 63.04 ± 4.33%, and 34.52 ± 2.66%, respectively. Thus, *AvL*-EtOH exhibited an inhibitory effect by suppressing the growth of these cells (Figure 2A). Interestingly, *AvL*-EtOH failed to exert any cytotoxic effects on WI-38 (normal lung cancer cells), as evaluated by the MTT assay (Figure 1B).

We performed phase-contrast microscopy to determine *AvL*-EtOH-mediated growth inhibition in A549 cells. Phase-contrast microscopy revealed that *AvL*-EtOH treatment results in the blebbing of the plasma membrane and decreases cell confluence, which are the indicators of possible apoptosis (Figure 2B). In addition, floating cells persistently appeared, suggesting that *AvL*-EtOH treatment reduced their adherence.

**Figure 3:**
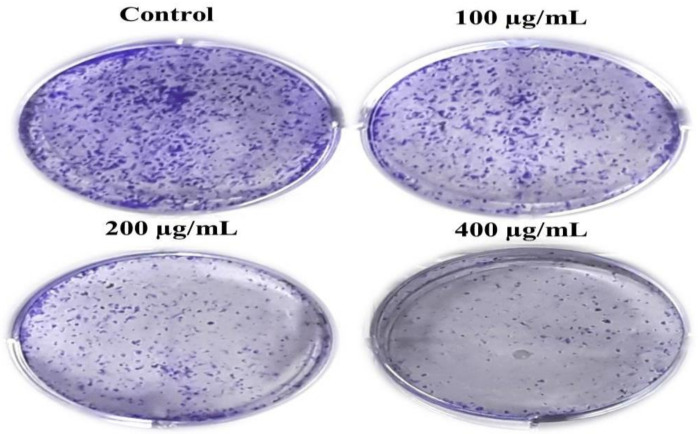
*AvL*-EtOH treatment resulted in the loss of clonogenic potential in NSCLC A549 cells. Representative images demonstrated the effect of *AvL*-EtOH on the loss of colony formation in A549 cells post *AvL*-EtOH-treatment.

Moreover, we performed the colony formation assay to determine the long-term effect of AvL-EtOH on A549 cell growth. Our findings show that *AvL*-EtOH treatment substantially decreased the number of colonies formed by the A549 cells in comparison to control cells. These results demonstrate that *AvL*-EtOH treatment suppressed the growth and clonogenic potential of the A549 cells (Figure 3A).

### 3.3. Nuclear Condensation in A549 Cells after AvL-EtOH Exposure

**Figure 4:**
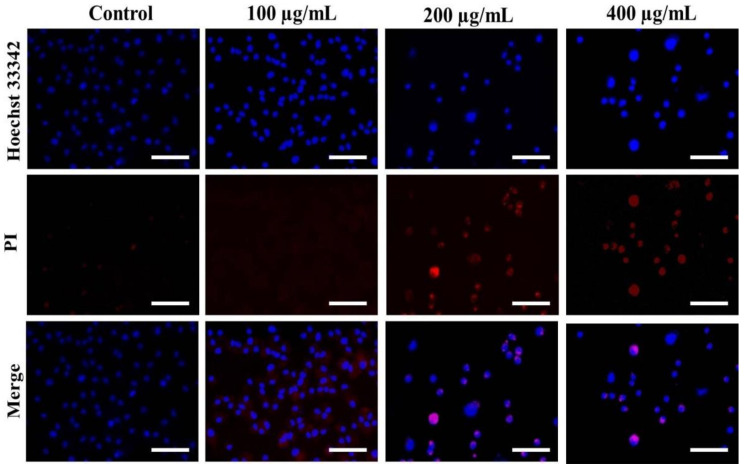
Evaluation of apoptosis instigated by varying concentrations of *AvL*-EtOH within A549 cells. Fluorescent photomicrographs exhibiting augmented levels of nuclear fragmentation and apoptosis within *AvL*-EtOH (100, 200 and 400 µg/mL)-treated A549 cells. Scale bar = 100 µm.

Hoechst staining was performed to determine whether the treatment of *AvL*-EtOH alters the nuclear morphology of A549 cells. *AvL*-EtOH treatment induces various alterations, such as nuclear shrinkage, chromatin condensation, and apoptotic body formation, in lung cancer cells, which are the characteristics of early and late apoptosis (Figure 4).

The stained nuclei in the control group were round and homogenous. Moreover, we used PI, a red fluorescent dye unable to pass through the plasma membrane of live cells, to stain the DNA of both apoptotic and necrotic cells. *AvL*-EtOH-treated cells exhibited substantial cell death (bright red fluorescence) (Figure 4).

### 3.4. AvL-EtOH Induced Apoptosis through Activation of Intrinsic as well as Extrinsic Pathway

**Figure 5:**
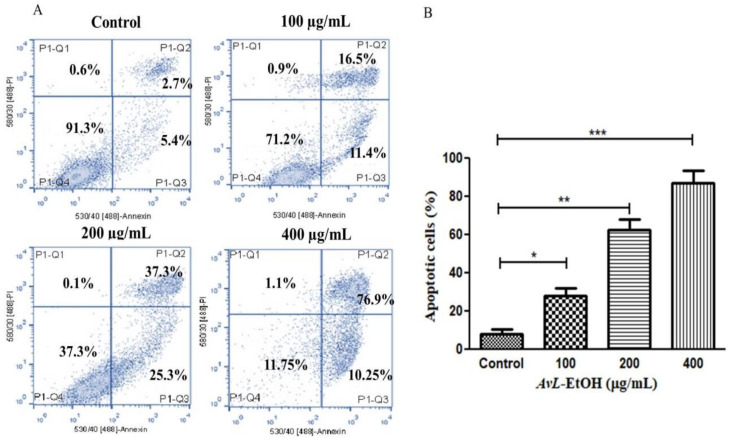
(**A**) Flow cytometric evaluation of apoptosis instigated within A549 cells post treatment with varying concentrations of *AvL*-EtOH (100, 200 and 400 µg/mL) and (**B**) graphical representation of flow cytometric data of apoptosis. * *p* < 0.05, ** *p* < 0.01 and *** *p* < 0.001.

The above findings were also validated by quantifying apoptosis activation by *AvL*-EtOH in A549 cells. It was noted that *AvL*-EtOH exhibited a dose-dependent increase in the number of apoptotic cells by 27.91 ± 3.31% (at 100 µg/mL), 62.61 ± 4.39% (at 200 µg/mL) and 87.15 ± 5.33% (at 400 µg/mL) as compared to the untreated control (8.13 ± 2.02%) (Figure 5A,B).

**Figure 6:**
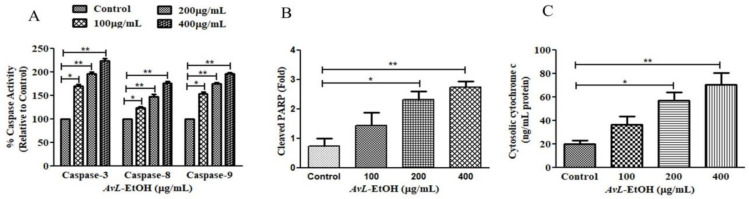
Efficacy of *AvL*-EtOH in modulating (**A**) the activities of different caspases, namely caspase-3, -8 and -9, (**B**) the level of cleaved PARP and (**C**) cytosolic level of cytochrome-c within A549 cells exposed to varying concentrations of *AvL*-EtOH (100, 200 and 400 µg/mL). * *p* < 0.05, ** *p* < 0.01.

To explore the underlying mechanism involved in *AvL*-EtOH-induced apoptosis, the activity of different caspases was determined (Figure 6A). The results show that *AvL*-EtOH (100, 200, and 400 μg/mL) increased the activities of caspase-8, -9, and -3 by 23.54 ± 2.81%, 47.39 ± 3.83%, and 77.02 ± 2.94%; 53.98 ± 3.36%, 74.85 ± 2.11%, and 96.53 ± 2.37%; and 70.03 ± 3.38%, 96.92 ± 2.98%, and 124.38 ± 3.73%, respectively. Moreover, *AvL*-EtOH dose-dependently induced PARP cleavage (Figure 6B), an indicator of apoptosis. The results also show an increase in the cytosolic cytochrome-c in *AvL*-EtOH-treated A549 cells (Figure 6C). These results suggest the involvement of the mitochondrial apoptosis pathway along with the extrinsic pathway in *AvL*-EtOH-treated A549 cells.

**Figure 7:**
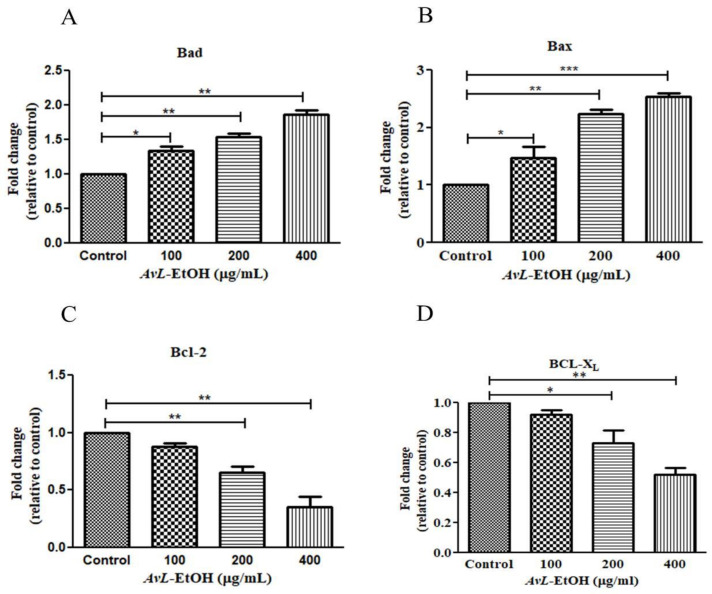
*AvL*-EtOH instigated changes in the gene expression of (**A**,**B**) pro-apoptotic (Bad and Bax), (**C**,**D**) anti-apoptotic (Bcl and Bcl-XL) proteins. * *p* < 0.05, ** *p* < 0.01and *** *p* < 0.001.

In addition, the real-time qPCR analysis revealed that *AvL*-EtOH (100, 200, and 400 μg/mL) markedly increased the expression of proteins involved in apoptosis onset (Bax and Bad) by 1.47 ± 0.15-, 2.24 ± 0.05-, and 2.53 ± 0.05-fold and 1.34 ± 0.04-, 1.54 ± 0.03-, and 1.86 ± 0.05-fold, and decreased the expression of anti-apoptotic genes (Bcl-XL and Bcl-2) by 0.82 ± 0.02-, 0.73 ± 0.07-, and 0.52 ± 0.03-fold and 0.82 ± 0.06-, 0.68 ± 0.15-, and 0.47 ± 0.14-fold, respectively, in A549 cells (Figure 7).

**Figure 8:**
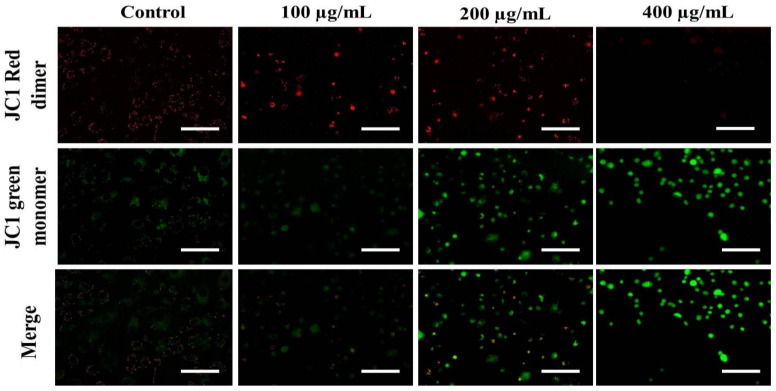
Fluorescent photomicrographs depicting the dissipation of ΔΨm within JC-1-stained A549 cells post *AvL*-EtOH exposure at concentrations of 100–400 µg/mL. Scale bar = 100 µm.

**Figure 9:**
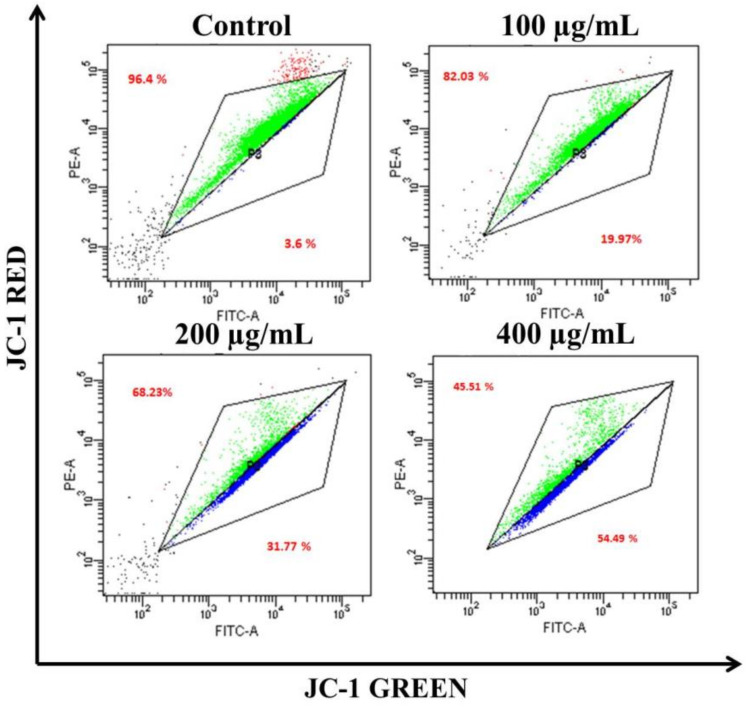
Flow cytometric dot plot elucidating the distribution of JC-1-stained cells (green: aggregated and blue: monomer form) indicating dissipation of ΔΨm within A549 cells post *AvL*-EtOH exposure (24 h) at concentrations of 100–400 µg/mL.

The loss of ΔΨm triggers cytochrome-c release from the mitochondria to the cytoplasm. Cyt c is regarded as the potent activator of the apoptotic caspase cascade. Thus, we determined the effects of *AvL*-EtOH on ΔΨm. Our data suggested that *AvL*-EtOH treatment decreased ΔΨm within the A549 cells (Figure 8 and Figure 9), verifying the involvement of mitochondria in *AvL*-EtOH-induced apoptosis.

**Figure 10:**
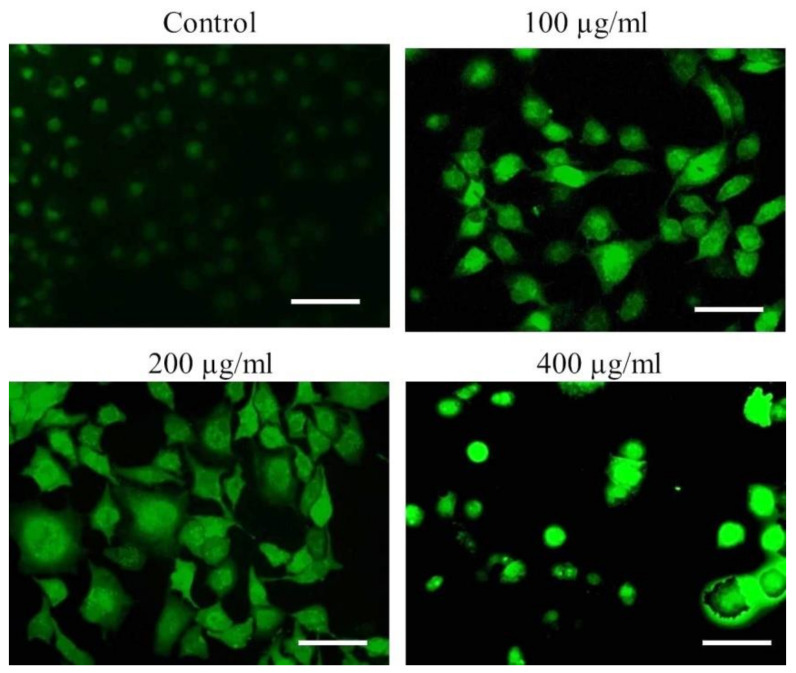
Qualitative assessment of ROS augmentation within *AvL*-EtOH-treated A549 cells. Fluorescent photomicrographs indicating enhanced DCF florescence within A549 cells treated with varying concentrations of *AvL*-EtOH (100, 200 and 400 µg/mL). Scale bar = 100 µm.

To further delineate the mechanics of *AvL*-EtOH-induced apoptosis, we assessed ROS generation through DCFH-DA staining. Fluorescence microscopy and flow cytometry demonstrated a dose-dependent increase in DCF fluorescence in the A549 cells, indicating enhanced ROS generation (Figure 10).

**Figure 11:**
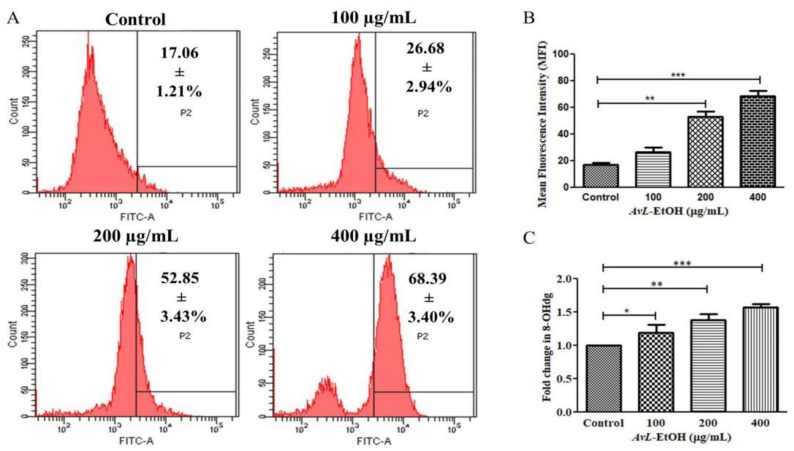
(**A**) Flow cytometric evaluation of ROS augmentation, (**B**) quantification of flow cytometry data indicating ROS augmentation (*n* = 3) and (**C**) fold change in the levels of 8-OHdG indicating DNA damage within A549 cells after treatment with varying concentrations of *AvL*-EtOH (100, 200 and 400 µg/mL). * *p* < 0.05, ** *p* < 0.01 and *** *p* < 0.001.

The mean fluorescence intensity of DCF-DA estimated through flow cytometry presented in Figure 10A,B demonstrates the efficacy of *AvL*-EtOH (100, 200, and 400 μg/mL) in escalating intracellular ROS by 26.68 ± 2.94%, 52.85 ± 3.43%, and 68.39 ± 3.40%, respectively, as compared with the control (17.06 ± 1.21%) (Figure 11A,B).

8-OHdG is a ubiquitous marker of oxidative stress and contributes significantly to carcinogenesis. As observed in Figure 11C, the fold change in the level of 8-OHdG was found to be 1.19 ± 0.09, 1.38 ± 0.07 and 1.57 ± 0.03, respectively, at 100, 200 and 400 µg/ mL in A549 lung cancer cells.

### 3.5. Modulation of Wnt/β-Catenin Signaling Pathway by AvL-EtOH

**Figure 12:**
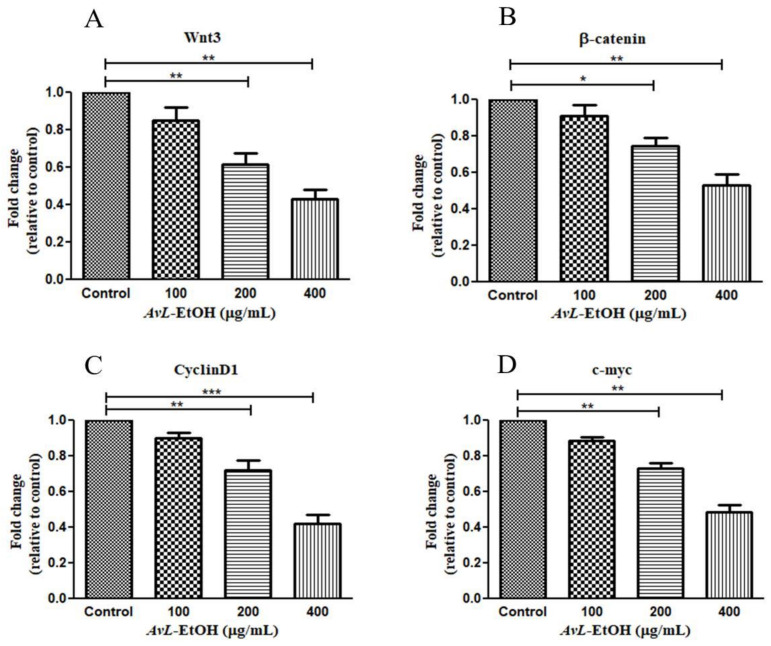
Effect of *AvL*-EtOH on Wnt signaling pathway and its downstream target genes. *AvL*-EtOH treatment inhibited the mRNA expression level of (**A**) Wnt3, (**B**) β-catenin, (**C**) cyclin D1 and (**D**) c-Myc in A549 NSCLC cells. * *p* < 0.05, ** *p* < 0.01 and *** *p* < 0.001.

The Wnt/β-catenin cascade is functionally activated with β-catenin’s translocation into the nucleus, activating the downstream target genes. The hyperactivation of Wnt3 critically modulates lung tumorigenesis by enhancing proliferation, migration, and invasion with the concomitant inhibition of apoptosis in cancer cells [25]. We, therefore, studied the effect of *AvL*-EtOH on the mRNA expression of the key components of this pathway. Wnt3 expression was significantly downregulated after treatment with *AvL*-EtOH. The Wnt3 expression levels were found to be 0.85 ± 0.05-, 0.61 ± 0.04-, and 0.43 ± 0.04-fold after treatment with 100, 200, and 400 μg/mL of *AvL*-EtOH, respectively, compared with those in the control. Similarly, *AvL*-EtOH inhibited the expression of β-catenin mRNA. The fold change in the expression of β-catenin mRNA was significantly reduced to 0.91 ± 0.04-, 0.74 ± 0.03-, and 0.53 ± 0.04-fold after exposure to 100, 200, and 400 μg/ mL *AvL*-EtOH, respectively, compared with the control. Furthermore, we evaluated the effect of *AvL*-EtOH on the expression level of β-catenin target protein mRNAs, such as cyclin D1 and c-myc, in the NSCLC cells. The mRNA expression of c-myc and cyclin D1 was substantially downregulated by 0.88 ± 0.06-, 0.72 ± 0.03-, 0.48 ± 0.03-, 0.90 ± 0.02-, 0.72 ± 0.04-, and 0.42 ± 0.05-fold with 100, 200, and 400 μg/mL *AvL*-EtOH treatment, respectively, in A549 NSCLC cells (Figure 12). Collectively, these results suggest that the downregulation of the Wnt/β-catenin cascade bestows antiproliferative- and apoptosis-inducing behavior on *AvL*-EtOH against NSCLC.

## 4. Discussion

Several signaling pathways are involved in the onset and progression of cancer. However, the exploration of novel and effective therapeutics for the clinical management of different cancers has surged globally [26]. Applying therapeutic agents with apoptosis-inducing potential is considered an effective intervention against various cancers [27,28]. Researchers across the globe are actively exploring natural compounds for developing novel chemotherapeutics for the effective clinical management of cancers primarily because crucial anticancer drugs that are being used presently, such as paclitaxel, docetaxel, vincristine, and vinblastine, are derived from natural products [29].

The members of the *Artemisia* L. genus constitute phytochemically essential plants exhibiting vivid biochemical and biological properties. These plants are distributed globally; however, they are commonly used to treat malaria, inflammation, and microbial and viral infections in China and other Asian countries [30,31]. *Artemisia kulbadica*, *A. diffusa*, *A. sieberi*, *A. santolina*, and *A. turanica* have shown anticancer effects on different cancer cells in vitro [32,33]. Furthermore, *A. vulgaris* considerably lowers the viability of human hepatocellular carcinoma HepG2 cells, indicating that *A. vulgaris* can induce apoptosis in HepG2 cells [34]. Moreover, *A. vulgaris* extracts have been effective against prostate and breast cancer cell lines and can sensitize breast cancer cells to tumor necrosis factor-related apoptosis-inducing ligands [35,36]. Nevertheless, the anticancer effect of *A. vulgaris* extract against NSCLC A549 cells remains unexplored, and this is why we aimed to investigate its potential against NSCLC A549 cells.

The ethanolic leaf extract of *A. vulgaris* L. showed the presence of several bioactive compounds. The chromatogram indicated the presence of different bioactive phytoconstituents such as terpenoids, flavonoids, carbohydrates, coumarins, amino acids, steroids, proteins, phytosterols, and diterpenes which could be responsible for their anticancer efficacy. The genus *Artemisia* has garnered significant phytochemical attention as it comprises various important medicinal plants. These plants possess various biological and chemical activities. Approximately 260 species of Artemisia have been inspected for their essential oils and secondary metabolites. Diverse classes of compounds such as flavonoids, sesquiterpenoids, diterpenoids, coumarins, isoprenylcoumaric acid derivatives, caffeoylquinic acids, sterols, phenoxychromene, acetophenone glucoside, phenylpropene, methyl jasmonate, and acetylenes have been identified as the main components [37,38,39]. It is noteworthy that multiple compounds from various classes have been studied for cytotoxic activity. It has been reported that the sesquiterpenoids are colorless and bitter compounds considered as the main compounds of the *Artemisia* species. Deoxyartemisinin, artemisinic acid, arteannuin-B and artemisitene were isolated from *Artemisia annua* L. In addition, the genus *Artemisia* is also a reservoir of coumarin-like compounds. Esculetin, scoparone, 7- methoxycoumarin, and dracumerin have been purified from *A. capillaris* and *A. dracunculus*; 6-methoxy-7,8-methylenedioxy coumarins from *A. indica*; isoarmenin and deoxylacarol from *A. armeniaca* Lam.; scopoletin from *A. princeps*; and scopoletin from *A. iwayomogi* [40].

Here, we demonstrated the anticancer and apoptotic effects of *AvL*-EtOH on A549 NSCLC cells. *AvL*-EtOH demonstrated potent cytotoxic effects on A549 cells, which could be associated with the inhibition of the Wnt signalling pathway. Uncontrolled proliferation and growth, as well as apoptosis evasion, are the major factors contributing to cancer initiation [41]. In this study, we showed that *AvL*-EtOH reduces the cellular growth of A549 cells, as revealed through the MTT assay. The clonogenic assay well supported these findings. Invasion and migration are the trademarks of cancer cell metastasis. Thus, agents that suppress the invasion and migration of cancer cells could be further explored to develop strategies for treating and preventing metastatic cancer. Our data demonstrated that *AvL*-EtOH treatment significantly inhibited A549 cell migration, which suggests the anti-metastatic potential of *AvL*-EtOH.

ROS generation leads to intensive DNA damage and induces DNA damage-dependent pathways. DNA damage beyond repair triggers cell death. A study reported that apoptosis is triggered by various DNA lesions [42,43,44]. *AvL*-EtOH-induced nuclear fragmentation and chromatin condensation were explicitly revealed through examination of the Hoechst/PI-stained A549 cells. Thus, *AvL*-EtOH treatment induces apoptotic cell death in NSCLC A549 cells. Moreover, flow cytometric data of the Annexin-V/FITC-stained cells demonstrated the appearance of apoptotic (early and late) and dead cells. These results are supported by the fluorescent micrographs of Hoechst/PI staining and an increased amount of cleaved PARP in the *AvL*-EtOH-treated A549 NSCLC cells.

Studies have suggested that ROS directly affect DNA damage, leading to apoptosis [45,46,47]. Furthermore, we observed the potential role of *AvL*-EtOH in the induction of ROS production, which is associated with numerous cellular events, resulting in apoptosis. Mitochondria are the main site for intracellular ROS generation [48], and the generation of excessive ROS results in mitochondrial dysfunction and the loss of ΔΨm due to the opening of mitochondrial permeability transition pores, providing a gateway for cyt c release [49]. We found significant differences in ΔΨm and the associated release of cyt c in response to *AvL*-EtOH treatment, which finally triggered the caspase cascade through caspase-3 activation, which is a key regulator of apoptosis progression, as it subsequently functionally activates crucial cellular proteins such as endonucleases [50]. Thus, *AvL*-EtOH substantially promotes ROS generation in A549 cells. ROS overproduction can induce oxidative stress, leading to apoptosis [51]. Thus, we can conclude that the induction of ROS production by *AvL*-EtOH might lead to apoptosis, thus confirming the anticancer properties of *AvL*-EtOH.

Our above results are in accordance with a previous observation where the ethanolic extract of *A. vulgaris* exerted a cytotoxic effect on HepG-2 with an IC50 value of 41.0 ± 1.0 μg/mL [22]. Furthermore, the essential oil of *A. vulgaris* exhibited anticancer efficacy against HL-60 leukemic cell line by inducing 94.8% apoptosis at 2.0 μg/mL via the release of cytochrome c and the activation of caspases-3, -9, and -8 [21]. In addition, a report demonstrated that the formulation of silver nanoparticles from the leaf extract of *A. vulgaris* showed substantial cytotoxicity against HeLa and MCF-7 cell lines via reducing their viability by 20% and 18%, respectively, at 140 μg/mL [23].

**Figure 13:**
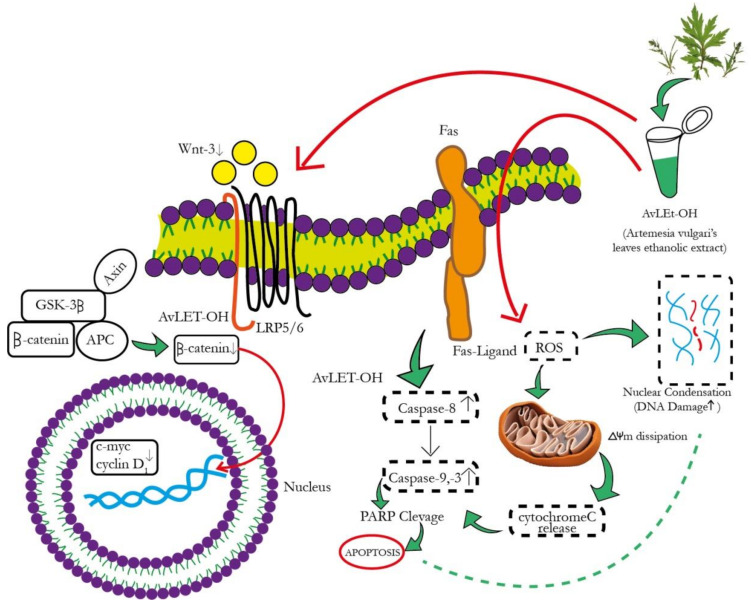
Schematic representation of the mechanism of action of *AvL*-EtOH against NSCLC. *AvL*-EtOH inhibits the proliferation of A549 lung cancer cells, leading to apoptosis and downregulation of the Wnt/β-catenin signaling pathway.

The canonical Wnt signaling cascade involves β-catenin-mediated activation of downstream pathways through different Wnt ligands. Intriguingly, Wnt3 and Wnt3a are crucial ligands upregulated in NSCLC [52,53]. These ligands subsequently bind to the FZD-7 receptor, activating the downstream β-catenin signaling pathway. *AvL*-EtOH treatment inhibits the mRNA expression of Wnt3 in lung cancer cells. The binding of Wnt3/3a to specific receptors was found to inhibit glycogen synthase kinase-3β (GSK-3β)-mediated phosphorylation of β-catenin through the removal of GSK-3β from the β-catenin degradation complex. The accumulation of β-catenin molecules in the cytoplasm triggers their translocation in the nucleus and culminates in the activation of genes related to cellular proliferation, stem cell characteristics (cyclin D1 and c-myc, respectively), metastasis (matrix metalloproteinases and E-cadherin), and angiogenesis (survivin and vascular endothelial growth factor) [54]. We observed that the *AvL*-EtOH-mediated inhibition of the β-catenin signaling pathway further decreases the mRNA expression of cyclin D1 and c-myc, thereby obstructing the growth and proliferation of human NSCLC cells. Thus, our study demonstrated that apoptosis in lung cancer cells treated with *AvL*-EtOH was mediated by inhibiting the Wnt/β-catenin signaling cascade. The proposed molecular mechanism of *AvL*-EtOH-mediated inhibition of the β-catenin signaling pathway in NSCLC is depicted in Figure 13.

## 5. Conclusions

This study demonstrated the antiproliferative and pro-apoptotic activities of *AvL*-EtOH against NSCLC A549 cells. *AvL*-EtOH induced caspase-mediated apoptosis, as evidenced by nuclear fragmentation and condensation, the loss of ΔΨm, cytochrome-c release, and PARP cleavage. These potential anticancer effects of *AvL*-EtOH are plausibly mediated through the downregulation of the canonical Wnt/β-catenin signaling cascade. Our findings provide a basis for studying the anticancer effects of various bioactive compounds *AvL*-EtOH against NSCLC cells. *AvL*-EtOH may serve as a novel adjuvant modality to the conventional chemotherapeutic regimen, with a significant therapeutic index and reduced side effects. However, in-depth mechanistic studies with in vivo models are warranted to confirm the preventive effect of *AvL*-EtOH against NSCLC.

## Figures and Tables

**Table 1:** Primer sequences used in the study.

Genes	Forward Sequence	Reverse Sequence
GAPDH	GAAATCCCATCACCATCTTCCAGG	GAGCCCCAGCCTTCTCCATG
Bax	GCCCTTTTGCTTCAGGGTTT	TCCAATGTCCAGCCCATGAT
Bad	CCTCAGGCCTATGCAAAAAG	AAACCCAAAACTTCCGATGG
Bcl2	GATTGTGGCCTTCTTTGAG	CAAACTGAGCAGAGTCTTC
Mcl-1	GGACATCAAAAACGAAGACG	GCAGCTTTCTTGGTTTATGG
Cyclin D1	CCGTCCATGCGGAAGATC	GAAGACCTCCTCCTCGCACT
c-myc	AGCGACTCTGAGGAGGAACAAG	GTGGCACCTCTTGAGGACCA
Wnt3	CGCTCAGCTATGAACAAGCA	AAAGTTGGGGGAGTTCTCGT
β-catenin	TCTGAGGACAAGCCACAAGATTACA	TGGGCACCAATATCAAGTCCAA
Bcl-XL	CAGAGCTTTGAACAGGTAG	GCTCTCGGGTGCTGTATTG

**Table 2:** Bioactive constituents recognized in the ethanolic extract of *A. vulgaris* L. leaves.

Peak No.	Retention Time	Area	Area (%)	Compound Identified	Molecular Formula
1.	4.464	71,441	0.83	4H-Pyran-4-one, 2,3-dihydro-3,5-dihydroxy-6-methyl	C_6_H_8_O_4_
2.	6.718	379,461	4.43	2,3,6,10,10-Pentamethyl-1-oxa-spiro(4,5)deca-3	C_14_H_22_O
3.	7.925	59,506	0.69	3-(Pyrrolidin-1-yl)cyclopent-2-en-1-one	C_9_H_13_NO
4.	8.382	561,175	6.55	2H-1-benzopyran-2-one	C_9_H_6_O_2_
5.	8.591	546,765	6.38	Guanosine	C_10_H_13_N_5_O_5_
6.	9.000	41,899	0.49	4H-1,2,3,6,7,8,9,9A-octahydroquinolizine-1,4,8-D	C_9_H_12_D_3_N
7.	9.086	27,165	0.32	2,4-Ditert-butylphenyl 5-hydroxypentanoate	C_19_H_30_O_3_
8.	9.241	104,843	1.22	Decanoic anhydride	C_20_H_38_O_3_
9.	10.063	128,609	1.50	1-(4-Methylphenyl)-1H-pyrrole	C_11_H_11_N
10.	10.942	1,664,345	19.42	1,3,4,5-Tetrahydroxycyclohexanecarboxylic acid	C_7_H_12_O_6_
11.	11.269	124,502	1.45	3alpha,7beta-Dihydroxy-5beta,6beta-epoxycholestane	C_27_H_46_O_3_
12.	11.356	40,194	0.47	1-Methyl-4-phenyl-1,2,3,6-tetrahydropyridine	C_12_H_15_N
13.	11.992	188,369	2.20	Nonylamine, N,N-di(allyl)	C_15_H_29_N
14.	12.207	44,211	0.52	(1R,3E,7E,11R)-1,5,5,8-Tetramethyl-12-oxabicyclo [9.1.0]dodeca-3,7-diene	C_7_H_12_O_6_
15.	12.299	40,009	0.47	6-Hydroxy-4,4,7a-trimethyl-5,6,7,7a-tetrahydrobenzofuran-2(4H)-	C_11_H_16_O_3_
16.	12.625	246,632	2.88	Arctiol	C_15_H_26_O_2_
17.	12.721	96,591	1.13	Neophytadiene	C_20_H_38_
18.	12.981	24,151	0.28	10,12-Tetradecadienal; Vernaldehyde	C_14_H_24_O
19.	13.074	45,010	0.53	2-Furanmethanol, tetrahydro-;Dihydropanaxacol	C_17_H_28_O_3_
20.	13.168	23,344	0.27	3,7,11,15-Tetramethylhexadec-2-en-1-yl acetate; Cetoleic acid	C_2_ O_22_H_42_
21.	13.414	123,198	1.44	2-(Dimethylamino)-N-(2,6-dimethylphenyl) acetamide; Lidocaine	C_14_H_22_N_2_O
22.	13.563	51,472	0.60	Limonene dioxide; Diosphenol	C_10_H_16_O_2_
23.	13.780	116,889	1.36	1H-Cyclopropa[a]naphthalene, 1a,2,3,5,6,7a,7b-octahydro-1,1,7,7a-tetramethyl-, 1aR-1aα,7α,7aα,7bβ	C_15_H_24_
24.	13.995	239,211	2.79	5,10-Diethoxy-2,3,7,8-tetrahydro-1H,6H-dipyrrolo[1,2-a:1’,2’-d]pyrazine; Rivastigmine	C_14_H_22_N_2_O_2_
25.	14.355	233,920	2.73	Scopoletin	C_10_H_8_O_4_
26.	14.840	124,027	1.45	Martynoside	C_31_H_40_O_15_
27.	14.980	29,151	0.34	Geranic acid	C_10_H_16_O_2_
28.	15.042	224,828	2.62	Deoxyartemisinin	C_15_H_22_O_4_
29.	15.222	111,627	1.30	Retinal	C_20_H_28_O
30.	15.341	88,859	1.04	5-oxatricyclo[4.4.0.01,4]decan-7-one; 4-Heptyloxyphenol	C_13_H_20_O_2_
31.	15.441	239,833	2.80	Phytol isomer	C_20_H_40_O
32.	15.749	182,147	2.13	Farnesol	C_15_H_26_O
33.	15.849	55,741	0.65	Cycloionone	C_13_H_20_O
34.	16.057	58,278	0.68	1H-2-Indenone,2,4,5,6,7,7a-hexahydro-3-(1-methylethyl)	
35.	16.339	25,000	0.29	1,8-Diazabicyclo[5.4.0]undec-7-en-11-one; Phenoxypropazine	C_9_H_14_N_2_O
36.	16.591	277,133	3.23	Cyperadione; Farnesoic acid	C_15_H_24_O_2_
37.	16.956	22,939	0.27	2-dimethylaminoethyl ester; Hexanoic acid	C_10_H_21_NO_2_
38.	18.447	64,492	0.75	N-cis-11-eicosaenoylethanolamine; 2-(Dimethylamino)ethyl vaccenoate	C_22_H_43_NO_2_
39.	19.034	48,587	0.57	Phthalic acid	C_24_H_38_O_4_
40.	20.269	119,663	1.40	1-Hexacosanol	C_26_H_54_O
41.	21.024	1,122,793	13.10	Erucamide; 13-Docosenamide	C_22_H_43_NO
42.	22.127	79,068	0.92	Chloroacetic acid	C_14_H_27_ClO_2_
43.	22.726	42,958	0.50	Octadecamethylcyclononasiloxane	C_18_H_54_O_9_Si_9_
44.	25.217	37,974	0.44	Vitamin E	C_29_H_50_O_2_
46.	25.760	92,327	1.08	4-(acetyloxy)-1-(1,5-dimethylhexyl)-3A,6,6,12A-tetramethyl-2,3,3A,3B,5A,6,7,8,9,11,12,12A-dodecahydro-1H-cyclopenta[A]cyclopropa[E]phenanthren-7-yl acetate	C_34_H_53_DO_4_
47.	27.639	102,832	1.20	Stigmasterone	C_29_H_46_O
48.	28.962	77,279	0.90	Stearoyldelicone	C_33_H_54_O_3_

## Data Availability

The data presented in this study are available in article.

## References

[1] Global Cancer Observatory, Cancer Factsheet. https://gco.iarc.fr/today/data/factsheets/cancers/15-Lung-fact-sheet.pdf.

[2] Batbold U., Liu J.J. (2022). Novel Insights of Herbal Remedy into NSCLC Suppression through Inducing Diverse Cell Death Pathways via Affecting Multiple Mediators. Appl. Sci..

[3] Hardy D., Cormier J.N., Xing Y., Liu C.C., Xia R., Du X.L. (2010). Chemotherapy-associated toxicity in a large cohort of elderly patients with non-small cell lung cancer. J. Thorac. Oncol..

[4] Holohan C., Van Schaeybroeck S., Longley D.B., Johnston P.G. (2013). Cancer drug resistance: An evolving paradigm. Nat. Rev. Cancer.

[5] Longley D.B., Johnston P.G. (2005). Molecular mechanisms of drug resistance. J. Pathol..

[6] Mukherjee P., Bahadur S., Harwansh R., Biswas S., Banerjee S. (2016). Paradigm shift in natural product research: Traditional medicine inspired approaches. Phytochem. Rev..

[7] Venkatesha S.H., Berman B.M., Moudgil K.D. (2011). Herbal medicinal products target defined biochemical and molecular mediators of inflammatory autoimmune arthritis. Bioorg. Med. Chem..

[8] Koni M., Pinnarò V., Brizzi M.F. (2020). The Wnt Signalling Pathway: A Tailored Target in Cancer. Int. J. Mol. Sci..

[9] Hafeez B.B., Siddiqui I.A., Asim M., Malik A., Afaq F., Adhami V.M., Saleem M., Din M., Mukhtar H. (2008). A dietary anthocyanidin delphinidin induces apoptosis of human prostate cancer PC3 cells in vitro and in vivo: Involvement of nuclear factor-kappaB signaling. Cancer Res..

[10] Du J.H., Zhang H.D., Ma Z.J., Ji K.M. (2010). Artesunate induces oncosis-like cell death in vitro and has antitumor activity against pancreatic cancer xenografts in vivo. Cancer Chemother. Pharmacol..

[11] Yoshikawa M., Shimada H., Matsuda H., Yamahara J., Murakami N. (1996). Bioactive constituents of Chinese natural medicines. I. New sesquiterpene ketones with vasorelaxant effect from Chinese moxa, the processed leaves of Artemisia argyi Levl. et Vant.: Moxartenone and moxartenolide. Chem. Pharm. Bull..

[12] Afsar S.K., Kumar K.R., Gopal J.V., Raveesha P. (2013). Assessment of antiinfammatory activity of Artemisia vulgaris leaves by cotton pellet granuloma method in Wistar albino rats. J. Pharm. Res..

[13] Jakovljević M.R., Grujičić D., Vukajlović J.T., Marković A., Milutinović M., Stanković M., Milošević-Djordjević O. (2020). In vitro study of genotoxic and cytotoxic activities of methanol extracts of *Artemisia vulgaris* L. and *Artemisia alba Turra*. S. Afr. J. Bot..

[14] Ahmad A., Tiwari R.K., Almeleebia T.M., Al Fayi M.S., Alshahrani M.Y., Ahmad I., Abohassan M.S., Saeed M., Ansari I.A. (2021). Swertia chirayita suppresses the growth of non-small cell lung cancer A549 cells and concomitantly induces apoptosis via downregulation of JAK1/STAT3 pathway. Saudi J. Biol. Sci..

[15] Tiwari R.K., Singh S., Gupta C.L., Bajpai P. (2021). Microglial TLR9: Plausible Novel Target for Therapeutic Regime Against Glioblastoma Multiforme. Cell Mol. Neurobiol..

[16] Sharma P.K., Bhardwaj R., Dwarakanath B.S., Varshney R. (2010). Metabolic oxidative stress induced by a combination of 2-DG and 6-AN enhances radiation damage selectively in malignant cells via non-coordinated expression of antioxidant enzymes. Cancer Lett..

[17] Ahmad A., Tiwari R.K., Saeed M., Ahmad I., Ansari I.A. (2022). Glycyrrhizin Mediates Downregulation of Notch Pathway Resulting in Initiation of Apoptosis and Disruption in the Cell Cycle Progression in Cervical Cancer Cells. Nutr Cancer..

[18] Ahmad A., Ansari I.A. (2021). Carvacrol Exhibits Chemopreventive Potential against Cervical Cancer Cells via Caspase-Dependent Apoptosis and Abrogation of Cell Cycle Progression. Anticancer Agents Med. Chem..

[19] Pandey B.P., Thapa R., Upreti A. (2017). Chemical composition, antioxidant and antibacterial activities of essential oil and methanol extract of *Artemisia vulgaris* and *Gaultheria fragrantissima* collected from Nepal. Asian Pac. J. Trop. Med..

[20] Van Nguyen Thien T., Tran L.T.K., Nhu N.T.T., Duc T.P., Do L.T.M., Tu D.D., That Q.T. (2018). A new eudesmane-type sesquiterpene from the leaves of *Artemisia vulgaris*. Chem. Nat. Compd..

[21] Ekiert H., Pajor J., Klin P., Rzepiela A., Ślesak H., Szopa A. (2020). Significance of *Artemisia vulgaris* L. (Common Mugwort) in the History of Medicine and Its Possible Contemporary Applications Substantiated by Phytochemical and Pharmacological Studies. Molecules.

[22] Saleh A.M., Aljada A., Rizvi S.A., Nasr A., Alaskar A.S., Williams J.D. (2014). In vitro cytotoxicity of *Artemisia vulgaris* L. essential oil is mediated by a mitochondria-dependent apoptosis in HL-60 leukemic cell line. BMC Complement. Altern. Med..

[23] Vahdati-Mashhadian N., Emami S., Oghazian M., Vosough R. (2009). The cytotoxicity evaluation of seven species of *Artemisia* on human tumor cell lines. Pharmacologyonline.

[24] Rasheed T., Bilal M., Iqbal H.M.N., Li C. (2017). Green biosynthesis of silver nanoparticles using leaves extract of *Artemisia vulgaris* and their potential biomedical applications. Colloids Surf. B Biointerfaces.

[25] Yip H.Y.K., Papa A. (2021). Signaling Pathways in Cancer: Therapeutic Targets, Combinatorial Treatments, and New Developments. Cells.

[26] Carneiro B.A., El-Deiry W.S. (2020). Targeting apoptosis in cancer therapy. Nat. Rev. Clin. Oncol..

[27] Lim B., Greer Y., Lipkowitz S., Takebe N. (2019). Novel Apoptosis-Inducing Agents for the Treatment of Cancer, a New Arsenal in the Toolbox. Cancers.

[28] Greenwell M., Rahman P.K. (2015). Medicinal Plants: Their Use in Anticancer Treatment. Int. J. Pharm. Sci. Res..

[29] Cragg G.M., Newman D.J. (2005). Plants as a source of anti-cancer agents. J. Ethnopharmacol..

[30] Fetrow C.W., Avila J.R. (2001). Professional’s Handbook of Complementary & Alternative Medicines.

[31] Gruenwald J., Brendler T., Jaenicke C. (2004). Physicians’ Desk Reference (PDR) for Herbal Medicines.

[32] Tang J., Zhao J.J., Li Z.H. (2015). Ethanol extract of *Artemisia sieversiana* exhibits anticancer effects and induces apoptosis through a mitochondrial pathway involving DNA damage in COLO-205 colon carcinoma cells. Bangladesh J. Pharmacol..

[33] Emami S.A., Vahdati-Mashhadian N., Vosough R., Oghazian M.B. (2009). The anticancer activity of five species of *Artemisia* on Hep2 and HepG2 cell lines. Pharmacologyonline.

[34] Sharmila K., Padma P.R. (2013). Anticancer activity of *Artemisia vulgaris* on hepatocellular carcinoma (HepG2) cells. Int. J. Pharm. Pharm. Sci..

[35] Abdelhamed S., Yokoyama S., Hafiyani L., Kalauni S.K., Hayakawa Y., Awale S., Saiki I. (2013). Identification of plant extracts sensitizing breast cancer cells to TRAIL. Oncol. Rep..

[36] Liu J.C., Deng T., Lehal R.S., Kim J., Zacksenhaus E. (2007). Identification of tumorsphere- and tumor-initiating cells in HER2/Neu-induced mammary tumors. Cancer Res..

[37] Abad M.J., Bedoya L.M., Apaza L., Bermejo P. (2012). The *artemisia* L. Genus: A review of bioactive essential oils. Molecules.

[38] Tang H.Q., Hu J., Yang L., Tan R.X. (2000). Terpenoids and flavonoids from *Artemisia* species. Planta Medica.

[39] Turi C.E., Shipley P.R., Murch S.J. (2014). North American *Artemisia* species from the subgenus *Tridentatae* (Sagebrush): A phytochemical, botanical and pharmacological review. Phytochemistry.

[40] Taleghani A., Emami S.A., Tayarani-Najaran Z. (2020). *Artemisia*: A promising plant for the treatment of cancer. Bioorganic Med. Chem..

[41] Roos W.P., Kaina B. (2006). DNA damage-induced cell death by apoptosis. Trends Mol. Med..

[42] Yang J., Zhao X., Tang M., Li L., Lei Y., Cheng P., Guo W., Zheng Y., Wang W., Luo N. (2017). The role of ROS and subsequent DNA-damage response in PUMA-induced apoptosis of ovarian cancer cells. Oncotarget.

[43] Srinivas U.S., Tan B.W.Q., Vellayappan B.A., Jeyasekharan A.D. (2019). ROS and the DNA damage response in cancer. Redox Biol..

[44] Bhardwaj M., Kim N.H., Paul S., Jakhar R., Han J., Kang S.C. (2016). 5-Hydroxy-7-Methoxyflavone Triggers Mitochondrial-Associated Cell Death via Reactive Oxygen Species Signaling in Human Colon Carcinoma Cells. PLoS ONE.

[45] Su J., Lai H., Chen J., Li L., Wong Y.S., Chen T., Li X. (2013). Natural borneol, a monoterpenoid compound, potentiates selenocystine-induced apoptosis in human hepatocellular carcinoma cells by enhancement of cellular uptake and activation of ROS-mediated DNA damage. PLoS ONE.

[46] Liang H.L., Sedlic F., Bosnjak Z., Nilakantan V. (2010). SOD1 and MitoTEMPO partially prevent mitochondrial permeability transition pore opening, necrosis, and mitochondrial apoptosis after ATP depletion recovery. Free Radic. Biol. Med..

[47] Lin X., Wei J., Chen Y., He P., Lin J., Tan S., Nie J., Lu S., He M., Lu Z. (2016). Isoorientin from *Gypsophila elegans* induces apoptosis in liver cancer cells via mitochondrial-mediated pathway. J. Ethnopharmacol..

[48] Zorov D.B., Juhaszova M., Sollott S.J. (2014). Mitochondrial reactive oxygen species (ROS) and ROS-induced ROS release. Physiol. Rev..

[49] Zhang Y., Zhang X., Huang J., Dong Q. (2015). Wnt signaling regulation of stem-like properties in human lung adenocarcinoma cell lines. Med. Oncol..

[50] Stewart D.J. (2014). Wnt signaling pathway in non-small cell lung cancer. J. Natl. Cancer Inst..

[51] Xu X., Sun P.L., Li J.Z., Jheon S., Lee C.T., Chung J.H. (2014). Aberrant Wnt1/β-catenin expression is an independent poor prognostic marker of non-small cell lung cancer after surgery. J. Thorac. Oncol..

[52] David M.D., Cantí C., Herreros J. (2010). Wnt-3a and Wnt-3 differently stimulate proliferation and neurogenesis of spinal neural precursors and promote neurite outgrowth by canonical signaling. J. Neurosci. Res..

[53] Okoye U.C., Malbon C.C., Wang H.Y. (2008). Wnt and Frizzled RNA expression in human mesenchymal and embryonic (H7) stem cells. J. Mol. Signal..

[54] Zhang M., Shi J., Huang Y., Lai L. (2012). Expression of canonical WNT/β-CATENIN signaling components in the developing human lung. BMC Dev. Biol..

